# Hochaltrige in Deutschland: Demografie, regionale Ungleichheit und Gesundheit – ein Überblick

**DOI:** 10.1007/s00103-026-04285-9

**Published:** 2026-08-18

**Authors:** Birgit Wolter, Kerstin Kammerer, Steffen Maretzke, Enno Nowossadeck

**Affiliations:** 1 Institut für Gerontologische Forschung e. V., Berlin, Deutschland; 2 Bundesinstitut für Bau‑, Stadt- und Raumforschung, Bonn, Deutschland; 3 Robert Koch-Institut, Gerichtstraße 27, 13347 Berlin, Deutschland

**Keywords:** Hochaltrigkeit, Lebenslage, Gesundheit, Regionale Unterschiede, Bevölkerungsprognose, Advanced age, Living situation, Health, Regional differences, Population forecast

## Abstract

Der Anteil Hochaltriger an der deutschen Bevölkerung nimmt kontinuierlich zu. Damit gewinnen auch ihre spezifischen Bedarfe, beispielsweise im Hinblick auf regionale Gesundheits- und Unterstützungsstrukturen, zunehmend an Bedeutung. Der Beitrag führt mit einem Überblick zur Lebenslage und gesundheitlichen Lage Über-80-Jähriger in das vorliegende Themenheft ein.

Zunächst wird die Bevölkerungsgruppe anhand demografischer Merkmale beschrieben. Unter den Hochaltrigen überwiegt deutlich der Frauenanteil; zudem zeigen sich geschlechtsspezifische Unterschiede hinsichtlich Einkommen, fernerer Lebenserwartung und Familienstand. Regional variiert ihr Bevölkerungsanteil zum Teil erheblich und steht im Zusammenhang mit dem jeweiligen regionalen Entwicklungspotenzial. Insbesondere in struktur- und einkommensschwachen Regionen ist der Anteil von Über-80-Jährigen an der Bevölkerung hoch.

Auf Basis der repräsentativen Studien Gesundheit 65+ (Robert Koch-Institut, RKI) und D80+ (ceres) werden zentrale Daten zur gesundheitlichen Lage Hochaltriger dargestellt. Thematisiert werden die subjektive Gesundheit, gesundheitliche Einschränkungen, Erkrankungen und Multimorbidität, Pflegebedürftigkeit sowie Demenz. Die Gesundheitsdaten machen die geschlechtsspezifischen Unterschiede und gesundheitliche Ungleichheit sichtbar und weisen auf Handlungsfelder hin, denen sich Kommunen bei der Sicherstellung gleichwertiger Lebensverhältnisse stellen müssen. Insbesondere strukturschwache Regionen stehen vor großen Herausforderungen, eine erforderliche Versorgungs- und Gesundheitsinfrastruktur für ein würdevolles Leben im Alter vorzuhalten.

**Zusatzmaterial online:**

Zusätzliche Informationen sind in der Online-Version dieses Artikels (https://doi.org/10.1007/s00103-026-04285-9) enthalten.

## Abstract (en)

The proportion of very elderly people in the German population is steadily increasing. As a result, their specific needs—for example, with regard to regional healthcare and support structures—are becoming increasingly important. This article introduces the current issue with an overview of the living conditions and health status of people aged 80 and over.

First, the population group is described based on demographic characteristics. Women clearly predominate among the very elderly; furthermore, gender-specific differences are evident in terms of income, remaining life expectancy, and marital status. Their share of the population varies regionally—in some cases considerably—and is linked to the respective region’s development potential. The proportion of very old people in the population is particularly high in structurally weak and low-income regions.

Based on the representative Gesundheit 65+ study (Robert Koch Institute, RKI) and D80+ study (ceres), key data on the health status of the very elderly are presented. The article addresses subjective health, health limitations, diseases and multimorbidity, need for care, and dementia. The health data, which also highlight gender-specific differences and health inequalities, point to fields of action that should be addressed in order to ensure equal living conditions. Structurally weak regions in particular face major challenges in providing the necessary care and health infrastructure for a dignified life in old age.

## Einleitung

Hochaltrige bilden einen zunehmend großen Anteil der deutschen Bevölkerung. Mit dem Älterwerden der Babyboomer-Generation wird die Bevölkerungsgruppe der 80-Jährigen und Älteren in den kommenden Jahren stetig wachsen. Damit gewinnen ihre spezifischen Bedarfe ebenfalls an Bedeutung, beispielsweise in Bezug auf regionale Gesundheits- oder Unterstützungsstrukturen. Hochaltrigkeit ist nicht per se mit Morbidität und Gebrechlichkeit verbunden, aber ihr Eintreten wird mit zunehmendem Alter wahrscheinlicher.

Dieser Beitrag gibt einen Überblick über die regionale Verteilung und gesundheitliche Lage von Hochaltrigen in Deutschland. Auf Grundlage von Daten des Statistischen Bundesamtes und Ergebnissen der Bevölkerungsprognose 2022 bis 2045 des Bundesinstituts für Bau‑, Stadt- und Raumforschung (BBSR; [1]) werden die aktuelle demografische Zusammensetzung der Altersgruppe, ihre regionale Verteilung sowie absehbare langfristige Veränderungen beschrieben. Dabei wird auf Herausforderungen eingegangen, die sich aus den unterschiedlichen regionalen Entwicklungspotenzialen und mit Blick auf die Sicherung gleichwertiger Lebensverhältnisse in den Regionen Deutschlands ergeben. Im anschließenden Abschnitt wird die gesundheitliche Lage Hochaltriger anhand ausgewählter Merkmale skizziert. Es werden Überblicksdaten zu subjektiver Gesundheit, gesundheitlichen Einschränkungen, Erkrankungen und Multimorbidität, Pflegebedürftigkeit und Demenz berichtet. Grundlage hierfür bilden die Surveys „Studie zur Gesundheit älterer Menschen in Deutschland (Gesundheit 65+)“ des Robert Koch-Instituts [2] und „Hohes Alter in Deutschland (D 80+)“ des Cologne Centre for Ethics, Rights, Economics, and Social Sciences of Health (ceres; [3]; Tab. 1) sowie eine Sonderaufbereitung von Daten zur Pflege durch das Forschungsdatenzentrum der Statistischen Ämter des Bundes und der Länder (FDZ; [4]).

**Tab. 1:** Surveys in Deutschland zur gesundheitlichen Lage im hohen Alter

	Studie zur Gesundheit älterer Menschen in Deutschland (Gesundheit 65+)	Studie Hohes Alter in Deutschland (Studie D80+)
Durchführung	Robert Koch-Institut	Cologne Centre for Ethics, Rights, Economics, and Social Sciences of Health, Deutsches Zentrum für Altersfragen (DZA)
Förderung	Bundesministerium für Gesundheit (BMG)	Bundesministerium für Familie, Senioren, Frauen und Jugend (BMFSFJ)
Ziel	Repräsentative Daten über die gesundheitliche Situation von Menschen im Alter von 65 Jahren und älter	Repräsentative Daten zur Lebenssituation und Lebensqualität von Menschen im Alter von 80 Jahren und älter
Themen	*Befragungen*: Gesundheitsstatus, COVID-19-bezogene Fragen, funktionelle Beeinträchtigungen, Gesundheitsversorgung, psychische Gesundheit, Gesundheitsverhalten, soziales Umfeld, soziodemografische Daten *Körperliche Untersuchungen:* anthropometrische Messungen (z. B. Körpergröße, Gewicht), Herz-Kreislauf-Funktionsmessungen, körperliche Funktionsfähigkeit, kognitive Funktionsfähigkeit, Anwendung von Nahrungsmitteln und Nahrungsergänzungsmitteln, Daten der gesetzlichen Krankenversicherung	Auswirkungen der Corona-Pandemie, Einkommen, Erkrankungen, Pflegebedürftigkeit und subjektive Gesundheit, Einsamkeit, soziale Eingebundenheit, digitale Teilhabe, Auswirkungen kognitiver Einschränkungen (Demenz), Alltagskompetenz und Wohnumfeld, Werte und Wünsche, Lebenszufriedenheit und Wohlbefinden
Methode	Längsschnittstudie Befragungen (online, telefonisch, persönlich) Körperliche Untersuchungen Arzneimittelinterview	Repräsentative Querschnittsbefragung (schriftlich und telefonisch)
Erhebungszeiträume	Gesamtzeitraum: 06.2021–04.2023 Basisbefragung 06.2021–04.2022, 3 Nachbefragungen nach 4, 8, 12 Monaten	11.2020–04.2021
Teilnehmende	Personen 65+, Wohnsitz in Deutschland, Privathaushalte und Pflegeeinrichtungen, ausreichende Deutschkenntnisse (Proxybefragungen waren möglich)	Personen 80+, Privathaushalte und Pflegeeinrichtungen, ausreichende Deutschkenntnisse Proxybefragungen waren möglich
Stichprobenumfang	3694 Teilnehmende, davon 2175 ab 80 Jahren	10.578 Teilnehmende (80–84: 59,1 %, 85–89: 26,9 %, 90+: 14 %)
Zugang	Einwohnermeldeamtsdaten, Zufallsstichprobe, postalische Kontaktaufnahme	Einwohnermeldeamtsdaten, Zufallsstichprobe, postalische Kontaktaufnahme
Weitere Informationen	Studie zur Gesundheit älterer Menschen in Deutschland (Gesundheit 65+): Zielsetzung, Konzeption und Durchführung, https://edoc.rki.de/handle/176904/11291, https://doi.org/10.25646/11662	Prussog-Wagner et al. [32] Methodenbericht. Deutschlandweite bevölkerungsrepräsentative Befragung hochaltriger Personen zu Lebensqualität und Wohlbefinden (D80+). Hrsg. Infas, Bonn https://ceres.uni-koeln.de/forschung/d80 DZA: Die 80+ Studie: https://www.dza.de/forschung/fdz/d80

## Hochaltrige: Demografischer Hintergrund

Im Jahr 2024 lebten in Deutschland 6,05 Mio. Hochaltrige^1^, das entspricht 7,2 % der Gesamtbevölkerung (vgl. hierfür und für die folgenden Angaben Tab. 2). Gegenüber 1990, dem Jahr der Wiedervereinigung, hat sich der Anteil der Hochaltrigen von damals um 3,8 % knapp verdoppelt. Gründe für diesen Anstieg bestehen insbesondere im Rückgang der vorzeitigen Sterblichkeit und damit im nachhaltigen Anstieg der Lebenserwartung sowie im niedrigen Geburtenniveau, das den Ersatz der Elterngeneration schon lange nicht mehr sichert [5, 6].

**Tab. 2:** Regionale Struktur- und Entwicklungsdaten zur Hochaltrigkeit

Kreistyp Wertschöpfungsniveau/alte und neue Länder/Deutschland	Hochaltrige							
	2011	2024	2011–2024	Frauenanteil 2024 (%)	2011	2024	2045	2011–2045
	Anzahl in 1000		%*		Bevölkerungsanteil (%)			(%)**
Sehr hohes Wertschöpfungsniveau	492	695	41,2	61,1	4,9	6,4	6,5	33,1
Hohes Wertschöpfungsniveau	326	472	44,6	60,4	4,9	6,8	7,9	59,5
Durchschnittliches Wertschöpfungsniveau	1054	1541	46,1	61,3	5,1	7,1	8,0	56,4
Niedriges Wertschöpfungsniveau	1538	2121	37,9	61,4	5,4	7,2	9,0	66,3
Sehr niedriges Wertschöpfungsniveau	858	1226	42,9	62,2	5,9	8,5	10,6	80,0
Westdeutschland	3396	4686	38,0	61,1	5,3	6,9	8,3	57,4
Ostdeutschland, inkl. Berlin	874	1369	56,7	62,5	5,5	8,5	9,7	76,2
Deutschland	4269	6055	41,8	61,4	5,3	7,2	8,5	60,9

* Entwicklung der Zahl der Hochaltrigen 2011 bis 2024 in %

** Entwicklung des Bevölkerungsanteils der Hochaltrigen 2011 bis 2045 in %

(Datenquelle: Statistik von Bund und Ländern, Datengrundlage: Fortschreibung des Bevölkerungsstandes; Bevölkerungsprognose des Bundesinstituts für Bau‑, Stadt- und Raumforschung (BBSR) 2045 (zensusbereinigt) [1])

**Abb. 1:**
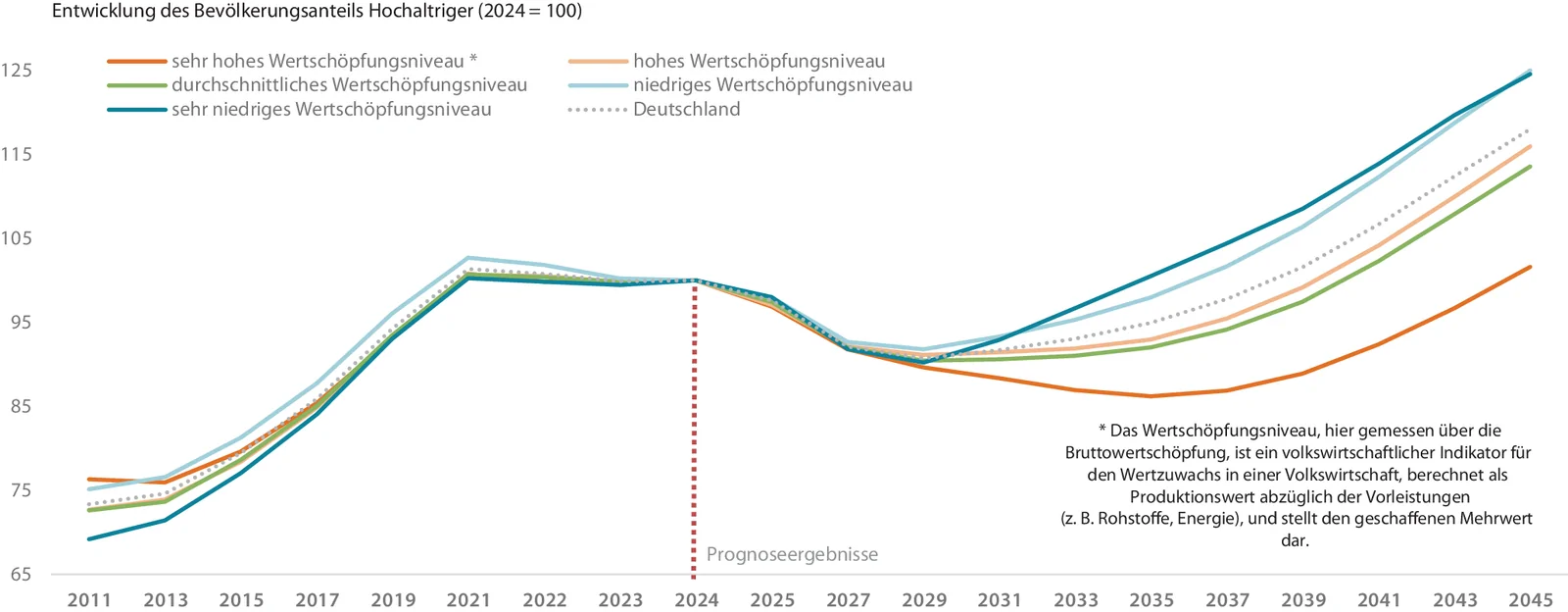
Regionale Entwicklungstrends der Hochaltrigkeit von 2011 bis 2045 (Datenquelle: Statistik von Bund und Ländern, Datengrundlage: Fortschreibung des Bevölkerungsstandes; Bevölkerungsprognose des Bundesinstituts für Bau‑, Stadt- und Raumforschung 2045/zensusbereinigt [1])

Die Zahl Hochaltriger wird künftig deutlich höher sein als gegenwärtig, bei tendenziell steigendem Bevölkerungsanteil. Die Bevölkerungsprognose des BBSR erwartet für das Jahr 2045 ca. 7,1 Mio. Hochaltrige (Frauen: 4,46 Mio.; Männer 2,64 Mio.; [7]). Der Anstieg verdeckt jedoch die Dynamik des Verlaufs. Wie Abb. 1 zeigt, hat sich der Bevölkerungsanteil der Hochaltrigen bis etwa 2021 kontinuierlich erhöht. Bedingt durch die Altersstruktur sinkt der Anteil bis zum Ende des Jahrzehnts, um danach bis 2045 weiter anzusteigen. Grund dafür ist, dass auf die geburtenschwachen Jahre um das Ende des Zweiten Weltkrieges 1945 herum Jahre mit sukzessiv steigenden Geburtenzahlen folgten [8, 9]. Letztere erreichen nunmehr die Altersgruppe 80 Jahre und älter. Ihre fernere Lebenserwartung unterscheidet sich nach Geschlecht: 80-jährige Frauen können im Durchschnitt von 9,6 Jahren ausgehen, Männer von 8,0 Jahren [10]. Dabei sind die Unterschiede zwischen den neuen und den alten Bundesländern geringfügig.

Das hohe Alter ist zu einem großen Teil weiblich: Knapp 2 Drittel der Hochaltrigen (61,4 %) sind Frauen. Von ihnen sind 62,2 % verwitwet, während 25,9 % in einer Ehe leben. Unter den hochaltrigen Männern stellt sich der Familienstand genau umgekehrt dar: 65,3 % der Männer leben in einer Ehe und 25,3 % sind verwitwet. Der Ausländeranteil unter den Hochaltrigen ist im Vergleich zur deutschen Gesamtbevölkerung relativ gering: 3,1 % der hochaltrigen Frauen und 4,4 % der Männer haben keine deutsche Staatsangehörigkeit [11].

Der relativ hohe Anteil alleinlebender hochaltriger Frauen spiegelt sich auch in den Haushaltsformen privater Haushalte wider. Von den 41 Mio. Haushalten in Deutschland sind 2,78 Mio. Einpersonenhaushalte, in denen jeweils eine Frau im Alter von 75 Jahren oder älter wohnt. Einpersonenhaushalte, in denen ein Mann lebt, kommen mit 850 Tsd. hingegen deutlich weniger häufig vor [12].

Einkommensdaten für Hochaltrige liegen nur spärlich vor. Auswertungen der Studie „Hohes Alter in Deutschland“ (D80+) haben diese Lücke teilweise geschlossen [13]. Sie zeigen, dass Armut bei Hochaltrigen häufiger auftritt (Armutsgefährdungsquote: 22 %) als in der Gesamtbevölkerung (15 %). Das Haushaltsnettoäquivalenzeinkommen^2^ in Privathaushalten beläuft sich bei Hochaltrigen auf 1885 € pro Monat und fällt damit deutlich geringer aus als in der Gesamtbevölkerung mit 2175 € [13]. Unterschiede treten auf zwischen Frauen (Nettoäquivalenzeinkommen: 1765 €) und Männern (2068 €) sowie zwischen Ost- (1758 €) und Westdeutschland (1923 €). Je höher der formale Bildungsgrad, umso höher ist auch bei Hochaltrigen das Einkommen: In der niedrigen Bildungsklasse beträgt das Nettoäquivalenzeinkommen 1426 €, in der mittleren 1859 € und in der hohen Bildungsklasse 2570 € (Unterschiede signifikant). In der niedrigen Bildungsklasse leben 42 % sowohl der Frauen als auch der Männer von einem Einkommen unterhalb der Armutsrisikogrenze, in der höchsten Bildungsklasse sind es 9 % der Frauen und 5 % der Männer.

Daten zum Vermögen im Alter zeigen, dass während des Erwerbsalters das höchste Vermögen aufgebaut wird, welches mit dem Übergang ins Rentenalter wieder abnimmt [14]. Westdeutsche wiesen im Jahr 2017 in allen Altersgruppen ein zum Teil eklatant höheres Vermögen als Ostdeutsche auf, auch in höheren Altersgruppen. Diese Ost-West-Unterschiede stiegen mit zunehmendem Lebensalter. Zwar wurde das Vermögen der Hochaltrigen nicht separat ausgewiesen [15]. Es ist aber davon auszugehen, dass sich diese Unterschiede auch bei den Hochaltrigen zeigen und sich seit 2017 kaum verändert haben.

## Regionale Strukturen und Trends der Hochaltrigkeit

Zwischen den neuen und alten Bundesländern bestanden 2024 [33] deutliche Unterschiede. In den neuen Bundesländern betrug der Anteil der Hochaltrigen 8,5 %, in den alten Bundesländern hingegen 6,9 %. Unterschiede im Anteil treten umso deutlicher hervor, je kleinräumiger die Regionen Deutschlands betrachtet werden.

Wirft man aus der regionalen Perspektive einen Blick auf die Zahl, Struktur und Entwicklung der Hochaltrigen im Zeitraum 2011 bis 2024, zeigen sich zwischen den Regionen Gemeinsamkeiten und Unterschiede. Während sich die Zahl der Hochaltrigen sowie ihr Bevölkerungsanteil bundesweit, also in allen Kreisen Deutschlands, erhöhte und in allen Regionen 2024 über 60 % der Hochaltrigen weiblich waren, zeigen sich in der Intensität der demografischen Alterung, hier gemessen am Bevölkerungsanteil der Hochaltrigen, z. T. enorme Unterschiede.

Aus der Erfahrung heraus [16], dass sich die demografische Situation einer Region in starker Abhängigkeit von ihrer wirtschaftlichen Lage gestaltet, wurden im Rahmen dieses Artikels die 400 Kreise Deutschlands – je nach Höhe ihres Wertschöpfungsniveaus (Bruttowertschöpfung^3^ je Erwerbstätigen) – 5 Kreistypen zugeordnet (Abb. 1 und Onlinematerial 1). So ist es möglich, die unterschiedlichen regionalen Entwicklungspotenziale bei dieser Analyse zu berücksichtigen. Kreise mit einem überdurchschnittlich hohen Wertschöpfungsniveau repräsentieren demnach strukturstarke Regionen, während Regionen mit einem niedrigen Wertschöpfungsniveau eher für Strukturschwäche stehen.

Tab. 2 zeigt zum einen, dass sich der Bevölkerungsanteil der Hochaltrigen von 2011 bis 2024 bundesweit sehr stark erhöht hat. Zum anderen wird deutlich, dass Kreise mit einem hohen Wertschöpfungsniveau 2024 einen niedrigeren Anteil der Hochaltrigen sowie von 2011 bis 2024 einen deutlich geringeren Anstieg des Anteils der Hochaltrigen an der Bevölkerung aufwiesen als Kreise mit einem niedrigen Wertschöpfungsniveau. Während sowohl strukturstarke als auch strukturschwache Kreise in diesem Zeitraum Wachstumsraten der Hochaltrigen von z. T. weit über 40 % hatten, zeigen sich beim Bevölkerungsanteil der Hochaltrigen deutlich ausgeprägtere regionale Unterschiede. Erhöhte sich der Bevölkerungsanteil der Hochaltrigen in den Kreisen mit einem sehr niedrigen Wertschöpfungsniveau von 2011 bis 2024 von 5,9 % auf 8,5 % (+ 44,1 %), so stieg dieser Anteil in den Kreisen mit einem sehr hohen Wertschöpfungsniveau lediglich um 30,6 % von 4,9 % auf 6,4 %. Die sehr strukturschwachen Kreise weisen also den höchsten Bevölkerungsanteil der Hochaltrigen und die stärkste Wachstumsdynamik des Bevölkerungsanteils der Hochaltrigen von 2011 bis 2024 auf.

Diese unterschiedliche regionale Wachstumsdynamik wird sich im Prognosezeitraum (2022 bis 2045) fortsetzen. Bis 2045 wird der Bevölkerungsanteil der Hochaltrigen deutschlandweit auf 8,5 % steigen, womit sich dessen Wachstumsdynamik (+17,1 %) deutlich verringert. Während der Bevölkerungsanteil der Hochaltrigen in den Kreisen mit einem sehr hohen Wertschöpfungsniveau im Prognosezeitraum aber lediglich auf 6,5 % anwachsen wird (+ 1,8 %), wird er in den Kreisen mit einem sehr niedrigen Wertschöpfungsniveau auf 10,6 % (+24,8 %), also überdurchschnittlich stark, steigen.

Verantwortlich für diese regionalen Unterschiede sind insbesondere Wanderungsbewegungen vor allem jüngerer Menschen, die überwiegend in strukturstärkere Regionen erfolgen. Diese verzeichneten in der Vergangenheit meist beachtliche Binnen- und/oder Außenwanderungsgewinne und weisen infolgedessen vergleichsweise niedrige Bevölkerungsanteile der Hochaltrigen auf [17]. Dagegen haben Wanderungsbewegungen die demografische Alterung in den strukturschwächeren Regionen noch weiter verstärkt, weil hier in der Regel eine relativ ungünstige Wanderungsbilanz vorliegt. Zum einen hatten diese Kreise in den letzten Jahren meist Binnenwanderungsverluste zu verzeichnen. Zum anderen partizipierten die strukturschwachen Kreise an den Außenwanderungsgewinnen, von denen in den letzten Jahren alle Kreise profitierten, meist deutlich weniger.

**Abb. 2:**
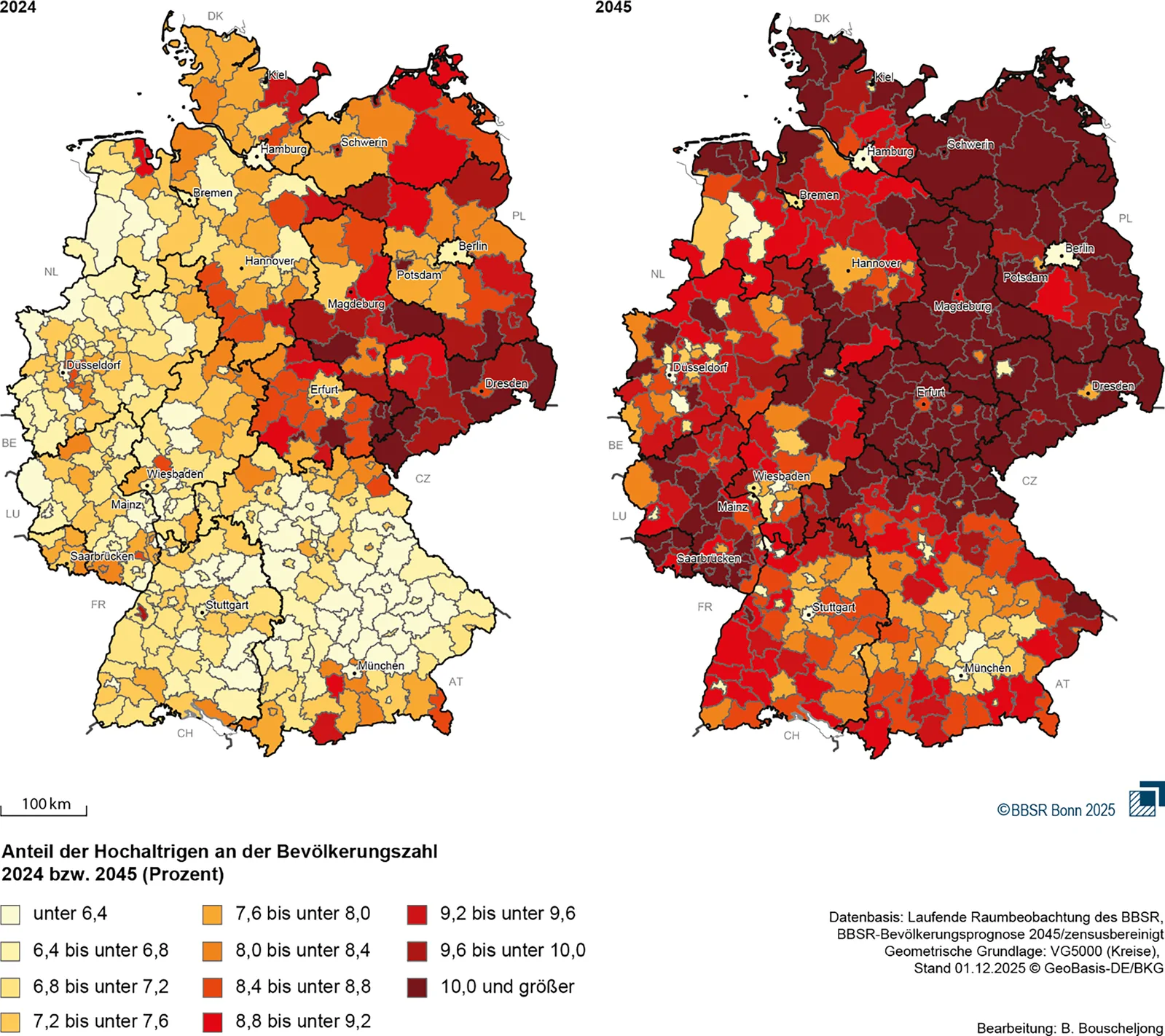
Bevölkerungsanteil der Hochaltrigen 2024 und 2045 in Deutschland auf Kreisebene

Nach der BBSR-Bevölkerungsprognose [1, 7] wird langfristig sowohl die Zahl der Hochaltrigen, als auch deren Bevölkerungsanteil bundesweit weiterwachsen (Abb. 2). Weil sich an den bekannten regionalen Mustern der Wanderungen im Prognosezeitraum wenig ändern wird [7], wird sich der Anteil der Hochaltrigen in den strukturstärkeren Regionen auch künftig in einem deutlich geringeren Maße als in den strukturschwächeren erhöhen.

Auf der Ebene der Kreise wird sich das Phänomen der Hochaltrigkeit voraussichtlich langfristig in die ostdeutschen Kreise verlagern. Unter den 50 Kreisen mit den höchsten Bevölkerungsanteilen Hochaltriger finden sich 2045 nach der BBSR-Prognose [1, 7] bei Anteilswerten von über 11,5 % nur 4 westdeutsche Landkreise (Ostholstein, Plön, Südwestpfalz und St. Wendel). In den ostdeutschen Landkreisen Spree-Neiße und Greiz werden die Anteile dann über 13,9 % liegen. Unter den 50 Kreisen mit den niedrigsten Anteilswerten liegen in Ostdeutschland dagegen nur Berlin und Leipzig. Die westdeutschen Stadtkreise Offenbach am Main und Frankfurt am Main weisen 2045 mit Anteilen von unter 4,7 % bundesweit die niedrigsten Werte auf. Insgesamt werden sich die regionalen Unterschiede bis 2045 erheblich vergrößern, vor allem aufgrund der höheren Wachstumsraten in den strukturschwächeren Kreisen. Damit verlagern sich auch die vielfältigen Herausforderungen der Hochaltrigkeit langfristig immer stärker in diese Regionen.

## Gesundheitliche Lage der Hochaltrigen

Die Darstellung der demografischen Struktur der Bevölkerung ab 80 Jahren und ihrer regionalen Verteilung in Deutschland zeigt: Die Regionen sind in unterschiedlichem Maß von der Alterung ihrer Bevölkerung betroffen. Die Lebenslagen von Hochaltrigen unterscheiden sich zwischen Männern und Frauen erheblich, vor allem hinsichtlich der Einkommen, der ferneren Lebenserwartung und des Familienstands. Mit Blick auf die gesundheitliche Lage Älterer interessiert im Folgenden unter anderem, ob sich auch hier Unterschiede zwischen den Geschlechtern abbilden sowie welche Gesundheitseinschränkungen oder Erkrankungen von besonderer Bedeutung und damit relevant für eine bedarfsgerechte, regionale Gesundheitsversorgung sind.

Ein hohes Alter bedeutet nicht unweigerlich, dass gesundheitliche Einschränkungen und ernsthafte Erkrankungen eintreten und zu Pflegebedürftigkeit führen; es wächst aber die Wahrscheinlichkeit. Mit zunehmendem Alter beeinflussen gesundheitliche Beeinträchtigungen vermehrt die Bewältigung des Alltags, die Lebenszufriedenheit und Lebensqualität. Insbesondere Multimorbidität kann dazu beitragen, dass die selbstständige Lebensführung herausfordernder wird und der Pflege- und Hilfebedarf zunimmt. Gleichzeitig verlaufen Alterungsprozesse individuell sehr unterschiedlich und sind nicht zuletzt mit dem sozioökonomischen Status assoziiert, der einen bedeutsamen Einfluss auf die Gesundheit hat [18]. Ein markantes Zeichen gesundheitlicher Ungleichheit sind Unterschiede in der Sterblichkeit [19]. Sozioökonomisch bedingte Ungleichheiten spiegeln sich dabei auch in regionalen Unterschieden der Sterblichkeit wider. Die Lebenserwartung differiert in den verschiedenen Regionen Deutschlands deutlich: Menschen in Regionen mit der höchsten sozioökonomischen Deprivation^4^ haben eine geringere Lebenserwartung als Menschen in den wohlhabendsten Regionen [19]. Der Unterschied beträgt für Frauen 4,3 Jahre, für Männer 7,2 Jahre und hat sich im Vergleich zu früheren Erhebungen von 2003/2005 vergrößert [19], ein Hinweis auf eine zunehmende gesundheitliche Ungleichheit in Deutschland [20].

### Subjektive Gesundheit

Die subjektive Gesundheit bzw. die selbst eingeschätzte Gesundheit gilt als ein Indikator für den objektiven Gesundheitszustand und u. a. als ein Prädiktor für die spätere Inanspruchnahme von Gesundheitsleistungen und für Mortalität [21]. Der selbst eingeschätzte Gesundheitszustand ist mit der aktiven Teilnahme am gesellschaftlichen Leben [21], dem Wohlbefinden, Gesundheitsverhalten und der psychischen Gesundheit assoziiert [22].

In der Studie „D80+“ wurde die subjektive Gesundheit durch die Frage: „Wie würden Sie Ihren Gesundheitszustand in den letzten 4 Wochen im Allgemeinen beschreiben?“, erhoben. Bei den Antwortalternativen wurde auf die mittlere Kategorie verzichtet, d. h., die Befragten mussten sich zwischen den Antwortmöglichkeiten „sehr gut“, „eher gut“, „eher schlecht“ oder „schlecht“ entscheiden [23]. Insgesamt war der Anteil der ab 80-Jährigen, die eine eher oder sehr gute Gesundheit angaben, mit 61,2 % relativ hoch, nahm aber mit zunehmendem Alter stetig ab.

Die Daten unterscheiden sich in verschiedenen Bevölkerungsgruppen: Frauen gaben häufiger einen (eher) schlechten Gesundheitszustand an als Männer. Auch Personen, die in stationären Pflegeeinrichtungen lebten, berichteten häufiger einen schlechten Gesundheitszustand als Ältere, die im eigenen Haushalt lebten [24]. Befragte in westdeutschen Bundesländern schätzten ihren Gesundheitszustand signifikant häufiger als „sehr gut“ oder „eher gut“ ein (62,2 %) als Befragte in den ostdeutschen Bundesländern (57,9 %; [24]). Zudem zeigen sich Unterschiede nach Bildungsabschlüssen: Eine sehr bzw. eher gute Gesundheit gaben über 2 Drittel (69,5 %) der Personen mit hoher Bildung, 61,3 % mit mittlerer Bildung und 55,4 % mit niedrigem Bildungsniveau an.

### Gesundheitliche Einschränkungen bei alltäglichen Aktivitäten, Erkrankungen und Multimorbidität

Im höheren Alter erschweren gesundheitliche Einschränkungen oft die eigenständige Bewältigung des Alltags. In der Studie 65+ wurden die Einschränkungen bei den basalen und instrumentellen Alltagsaktivitäten erhoben [25]. Als basale Alltagsaktivitäten zählen Nahrungsaufnahme, Aufstehen oder Setzen, An- und Ausziehen, Toilettenbenutzung und Körperhygiene. Instrumentelle Alltagsaktivitäten sind die Zubereitung von Mahlzeiten, Telefonnutzung, Erledigung von Einkäufen, leichte oder gelegentliche schwere Hausarbeit, Organisation der Medikamenteneinnahme, Erledigung finanzieller und alltäglicher Verwaltungsangelegenheiten. Bei der Verrichtung basaler Alltagsaktivitäten waren 27 % der ab 80-jährigen Frauen und 15,3 % der Männer dieser Altersgruppe eingeschränkt, bei instrumentellen Alltagsaktivitäten waren es 48,7 % der Frauen und 29,7 % der Männer. Eine ausführliche Darstellung der körperlichen Funktionsfähigkeit Hochaltriger erfolgt in dem Beitrag von Nowossadeck et al. in diesem Themenheft.

Nicht nur gesundheitliche Einschränkungen, sondern auch Krankheiten treten mit zunehmendem Alter häufiger auf. Mit 98 % sind nahezu alle Hochaltrigen von mindestens einer Erkrankung betroffen [22]. Das Vorliegen von 2 oder mehr Krankheiten und Gesundheitsproblemen wird in den Studien „Gesundheit 65+“ und „D80+“ als Multimorbidität bezeichnet, hierzu zählen u. a. auch seelische Erkrankungen, Sehstörungen oder Schwerhörigkeit [24, 25]. Ähnlich wie bei der subjektiven Gesundheit werden auch bei Multimorbidität signifikante Unterschiede zwischen den Geschlechtern, Bildungsgruppen und Regionen sichtbar.

In der Studie D80+ berichteten 90,2 % der Über-80-Jährigen, an 2 oder mehr Krankheiten oder Gesundheitsproblemen zu leiden. Im Durchschnitt bestanden 4,7 ärztlich behandelte Erkrankungen, dabei waren Frauen deutlich stärker betroffen [24]. Die am häufigsten angegebenen Erkrankungen der Hochaltrigen waren Bluthochdruck (65,1 %), Gelenk- oder Knochenerkrankungen (55 %) und Rückenschmerzen (44,6 %). Die Studie „Gesundheit 65+“ beschreibt einen sehr deutlichen Unterschied zwischen den Geschlechtern bei insgesamt niedrigeren Prävalenzen als die Studie „D80+“: Demnach waren 82,4 % der ab 80-jährigen Frauen und 70,9 % der Männer dieser Altersgruppe von Multimorbidität betroffen [25]. Die unterschiedlichen Ergebnisse der beiden Surveys werden u. a. auf methodische Unterschiede bei den Erhebungen zurückgeführt [25].

Die Studie „D80+“ zeigt zudem, dass sich Multimorbidität nach Bildungsgruppen unterscheidet: Während Menschen mit niedriger formaler Bildung durchschnittlich 5 Erkrankungen aufweisen, sind es bei hochgebildeten Menschen 4,4 Erkrankungen [24]. Wie schon bei der subjektiven Gesundheit zeigen sich auch hier bedeutsame Ost-West-Unterschiede: Hochaltrige in Westdeutschland weisen statistisch signifikant weniger Erkrankungen auf als in den östlichen Bundesländern (Westdeutschland: 4,7, Ostdeutschland: 4,9 Erkrankungen; [24]).

Krankheiten gehen häufig mit Schmerzen einher, die Lebensqualität und Alltagsaktivitäten stark einschränken können. In der Studie „Gesundheit 65+“ wurden Schmerzen und Schmerzintensität erfragt. Wenn starke oder sehr starke Schmerzen länger als 6 Wochen anhielten, wurden sie als chronische Schmerzen erfasst [25]. Nach dieser Definition gaben 22,2 % der ab 80-jährigen Frauen und 14,9 % der Männer dieser Altersgruppe an, unter chronischen Schmerzen zu leiden.

## Pflegebedürftigkeit im hohen Alter

Im Jahr 2023 gab es in Deutschland 2,1 Mio. Pflegebedürftige (Tab. 3). Von den Über-80-Jährigen hatten 2023 ca. 50 % einen Pflegegrad. Das Risiko der Pflegebedürftigkeit wächst mit steigendem Alter rapide von 35 % unter den 80- bis Unter-85-Jährigen auf 97 % bei den Über-95-Jährigen ([26], eigene Berechnung). Im selben Jahr waren 69,6 % der Pflegebedürftigen 80 Jahre alt oder älter, wobei über ein Drittel dieser hochaltrigen Pflegebedürftigen (34,4 %) in Pflegeheimen betreut wurde, das heißt, sie erhielten Dauer‑, Kurzzeit‑, Tages- oder Nachtpflege. Die große Mehrheit der hochaltrigen Pflegebedürftigen, nämlich 81 %, wurde in Privathaushalten gepflegt – überwiegend von oft selbst schon älteren Familienangehörigen. Familien werden in diesem Zusammenhang auch als „größter Pflegedienst in Deutschland“ bezeichnet [27, 28].

**Tab. 3:** Ausgewählte Indikatoren zur Regionalstruktur der Pflegebedürftigkeit Hochaltriger 2023

Regionstyp* Wertschöpfungsniveau**/West- und Ostdeutschland/Deutschland	Pflegebedürftige (PB)	Hochaltrige Pflegebedürftige					Anteil hochaltrige PB an den PB insg.	Anteil hochaltrige PB Frauen an den PB insg.
		Insgesamt		In ambulanter Pflege	In Pflegeheimen	Weibliche PB je 100 hochaltrige Frauen		
	In 1000		Je 100 Hochaltrige				%	
Sehr hohes Wertschöpfungsniveau	245	171	19,8	9,9	9,9	23,7	69,8	72,4
Hohes Wertschöpfungsniveau	259	181	21,2	10,5	10,8	25,3	70,1	72,5
Durchschnittliches Wertschöpfungsniveau	318	220	22,3	10,8	11,5	26,4	69,2	72,9
Niedriges Wertschöpfungsniveau	998	695	25,6	12,9	12,7	30,3	69,7	73,1
Sehr niedriges Wertschöpfungsniveau	258	179	28,8	15,8	13,0	33,9	69,3	73,9
Westdeutschland	1551	1082	23,1	11,3	11,8	27,5	69,8	72,8
Ostdeutschland, inkl. Berlin	526	364	26,9	15,0	11,9	31,6	69,2	73,6
Deutschland	2077	1446	23,9	12,1	11,8	28,4	69,6	73,0

* Die Abgrenzung des Regionstyps nach Höhe des Wertschöpfungsniveaus wurde hier auf Basis von Raumordnungsregionen (in der Regel das Aggregat mehrerer Kreise) vorgenommen. Die zugrunde liegenden Daten aus der Pflegestatistik, die aus Datenschutzgründen nur für Raumordnungsregionen verfügbar sind, wurden im Rahmen einer Sonderaufbereitung durch ein Forschungsdatenzentrum der Statistischen Ämter des Bundes und der Länder bereitgestellt

Übersicht der Regionen nach Wertschöpfungsniveau vgl. Onlinematerial 2

** Das Wertschöpfungsniveau, hier gemessen über die Bruttowertschöpfung, ist ein volkswirtschaftlicher Indikator für den Wertzuwachs in einer Volkswirtschaft, berechnet als Produktionswert abzüglich der Vorleistungen (z. B. Rohstoffe, Energie), und stellt den geschaffenen Mehrwert dar

(Datenquelle: FDZ 2025 [4]; eigene Berechnungen)

Die Pflegebedürftigkeit der Männer und Frauen weist bundesweit erhebliche Unterschiede auf. Zum einen sind aufgrund des höheren Anteils der Frauen an den Hochaltrigen mehr Frauen als Männer von Pflegebedürftigkeit betroffen. So waren 2023 über 2 Drittel aller Pflegebedürftigen weiblich. Bei den hochaltrigen Pflegebedürftigen waren es sogar 73,0 %. Zum anderen sind hochaltrige Frauen pflegebedürftiger als Männer dieser Altersgruppe. Während von den hochaltrigen Männern lediglich 16,8 % pflegebedürftig waren, lag dieser Wert bei den Frauen bei 28,4 %.

Die regionalen Unterschiede, die sich beim Bevölkerungsanteil der Hochaltrigen beobachten lassen, zeigen sich auch bei der regionalen Verteilung der Pflegebedarfs. So konzentrieren sich die Herausforderungen im Bereich der Pflege Hochaltriger vor allem auf die Regionen mit niedrigem Wertschöpfungsniveau (Tab. 3). Diese Regionen weisen den höchsten Frauenanteil hochaltriger Pflegebedürftiger auf und der Anteil der Hochaltrigen, die ambulante Pflege oder Betreuung in Pflegeheimen in Anspruch nehmen, erreicht bundesweite Höchstwerte. Im Gegensatz dazu liegen die Vergleichswerte in den strukturstärkeren Regionen erheblich niedriger. Beispielhaft für diese regionalen Unterschiede steht hier der Anteil der hochaltrigen Pflegebedürftigen an der Gesamtzahl der Pflegebedürftigen. Während beispielsweise in den strukturschwachen Regionen Vorpommern, Anhalt-Bitterfeld-Wittenberg, Mecklenburgische Seenplatte, Emsland und Nordthüringen über 33 % der Hochaltrigen pflegebedürftig waren, lag dieser Anteil in den meist strukturstärkeren Regionen Ostwürttemberg, Ingolstadt, Allgäu, Bodensee-Oberschwaben, Neckar-Alb, Oberland und München unter 18 %. Diese Werte signalisieren, dass sich im Bereich der Pflege die regional unterschiedlichen Herausforderungen auch in der Ost-West-Dimension zeigen.

### Demenz

Demenzielle Erkrankungen sind ein häufiger Grund für Pflegebedürftigkeit im Alter [29]. Eine Demenz entwickelt sich meist schleichend über einen längeren Zeitraum und führt zu wachsenden Einschränkungen und Abhängigkeiten der Betroffenen bis hin zu Pflegebedürftigkeit mit hohem Unterstützungsbedarf [30]. Etwa 2 Drittel der Erkrankten leiden unter Alzheimer-Demenz, gefolgt von vaskulärer Demenz [31]. Die Prävalenz von Demenzerkrankungen nimmt mit dem hohen Alter stark zu, wie eine Auswertung von Versichertendaten der Gesetzlichen Krankenversicherungen (GKV) aus dem Jahr 2022 zeigt: Während in der Altersgruppe „80 bis unter 85 Jahren“ der Anteil der an Demenz Erkrankten etwa 10 % beträgt, steigt er bei den Über-95-Jährigen auf nahezu ein Drittel [31]. Frauen sind in allen höheren Altersgruppen stärker von Demenz betroffen als Männer. Zudem zeigen sich in Deutschland (altersübergreifend) deutliche regionale Unterschiede: Sowohl die ostdeutschen Bundesländer als auch das Saarland und einige Regionen von Nordrhein-Westfalen weisen überdurchschnittlich hohe Prävalenzen auf. Die regionale Verteilung folgt dem Muster der bundesweit ungleich verteilten sozialen Deprivation, die mit bestimmten Risikofaktoren für Demenz assoziiert wird [31]. Die Versorgung von demenziell Erkrankten geschieht überwiegend ambulant bzw. durch Angehörige. Der Studie „D80+“ zufolge leben über 2 Drittel der an Demenz erkrankten Hochaltrigen in Privathaushalten (69,6 %), knapp 20 % werden in Pflegeeinrichtungen betreut und etwa 12 % leben in Wohnmodellen, wie Mehrgenerationenhäusern oder Wohnpflegegruppen [30]. Hierbei handelt es sich meist um schwer erkrankte Personen. Etwa 57 % der demenziell Erkrankten versterben in einer Pflegeeinrichtung [30]. Risiko- und Schutzfaktoren für Demenz werden in dem Beitrag von Wittmann et al. in diesem Themenheft dargestellt.

## Limitationen

Die Studien „Gesundheit 65+“ und „D80+“ wurden herangezogen, da sie in Pflegeeinrichtungen lebende Personen miteinbeziehen. Diese wurden in beiden Studien jedoch seltener erreicht als in Privathaushalten lebende Personen [25, 32]. In der Studie Gesundheit 65+ wurden Personen in sozial benachteiligten Wohngegenden seltener erreicht und in beiden Studien wurden Personen ohne ausreichende Deutschkenntnisse ausgeschlossen [25, 32].

## Fazit

Die zunehmende Bedeutung von Hochaltrigkeit als gesellschaftliches Phänomen resultiert aus der demografischen Alterung, die sich aus dem Anstieg der Lebenserwartung und dem schon lange sehr niedrigen Geburtenniveau ergibt. Dabei haben Frauen eine höhere Lebenserwartung als Männer und sind demzufolge häufiger in der Hochaltrigengruppe vertreten. Sie sind im hohen Alter häufiger verwitwet und leben öfter in Einpersonenhaushalten als Männer. Hochaltrige Frauen sind im Durchschnitt materiell schlechter gestellt als gleichaltrige Männer. Zugleich sind gerade alte Menschen mit geringem Einkommen und niedrigem Bildungsstatus stärker von gesundheitlichen Einschränkungen und Erkrankungen betroffen. Kritisch anzumerken ist hierbei, dass die Datenlage zur Gesundheit Hochaltriger für Menschen mit Migrationshintergrund und für Pflegebedürftige unzureichend ist, sodass die Gesundheitsbelastungen in dieser Altersgruppe sowie die gesundheitliche Ungleichheit vermutlich unterschätzt werden.

Der Hochaltrigenanteil an der Bevölkerung unterliegt einer erheblichen Dynamik: Ab 2030 ist in den meisten Regionen eine weitere Zunahme zu erwarten. Die regionalen Unterschiede sind groß und verstärken sich langfristig weiter, vor allem zulasten der strukturschwächeren Regionen. Gerade in diesen Regionen kumulieren damit die Herausforderungen bezüglich der Sicherung gleichwertiger Lebensverhältnisse weiter. Im Jahr 2045 wird der Bevölkerungsanteil Hochaltriger in den strukturschwächeren Regionen mindestens 50 % über den Werten der strukturstärkeren Regionen liegen. Daher müssen sich gerade diese Regionen mit begrenzten Ressourcen auf steigende Bedarfe bei der gesundheitlichen und pflegerischen Versorgung von Hochaltrigen einstellen. Im Zuge dieser Entwicklung werden auch die Anforderungen an die Bereitstellung einer leistungsfähigen Infrastruktur im Gesundheits- und Pflegebereich steigen, wenn für alle Regionen gleichwertige Voraussetzungen für ein gutes Leben im Alter gesichert werden sollen.

## Supplementary Information

Grafiken zum Hochaltrigenanteil und zu den verwendeten Kreis- und Regionstypen des Wertschöpfungsniveaus in Deutschland

## Data Availability

Die dargestellten Daten sind unter den angegebenen Quellen öffentlich zugänglich oder auf begründete Anfrage beim Autorenteam erhältlich.

## Footnotes

1 In diesem Beitrag gelten Personen als hochaltrig, die 80 Jahre oder älter sind. Andere Altersabgrenzungen werden vorgenommen, wenn die Datenquelle es erfordert, bspw. im Mikrozensus (75 Jahre und älter).

2 Mithilfe des Indikators „Haushaltsnettoäquivalenzeinkommen“ lässt sich die Einkommenssituation von Haushalten unterschiedlicher Größe und Zusammensetzung vergleichen. Dazu wird das verfügbare Haushaltseinkommen unter Berücksichtigung der Zahl und des Alters der Haushaltsmitglieder gewichtet und rechnerisch auf die einzelnen Personen verteilt.

3 Die Bruttowertschöpfung ist ein volkswirtschaftlicher Indikator für den Wertzuwachs in einer Volkswirtschaft, berechnet als Produktionswert abzüglich der Vorleistungen (z. B. Rohstoffe, Energie), und stellt den geschaffenen Mehrwert dar.

4 Sozioökonomische Deprivation wird mit dem German Index of Socioeconomic Deprivation (GISD) erfasst. Der GISD umfasst 9 räumlich aggregierte Einzelindikatoren, um 3 Kerndimensionen sozioökonomischer Ungleichheit (Bildung, Beschäftigung, Einkommen) abzubilden. Es wird ein Gesamtindex gebildet [23].
