# Supplementary material for: The very elderly in Germany: demography, regional inequalities, and health—an overview
Source: Bundesgesundheitsblatt Gesundheitsforschung Gesundheitsschutz. 2026 Aug 18;69(9):981–90. [Article in German] doi: 10.1007/s00103-026-04285-9 (PMC13562148; doi:10.1007/s00103-026-04285-9)
Supplement: Supplementary file 1 [file 103_2026_4285_MOESM1_ESM.pdf]

Onlinematerial 1: Bevölkerungsanteil der Hochaltrigen 2023 und 2045 in Deutschland auf Kreisebene

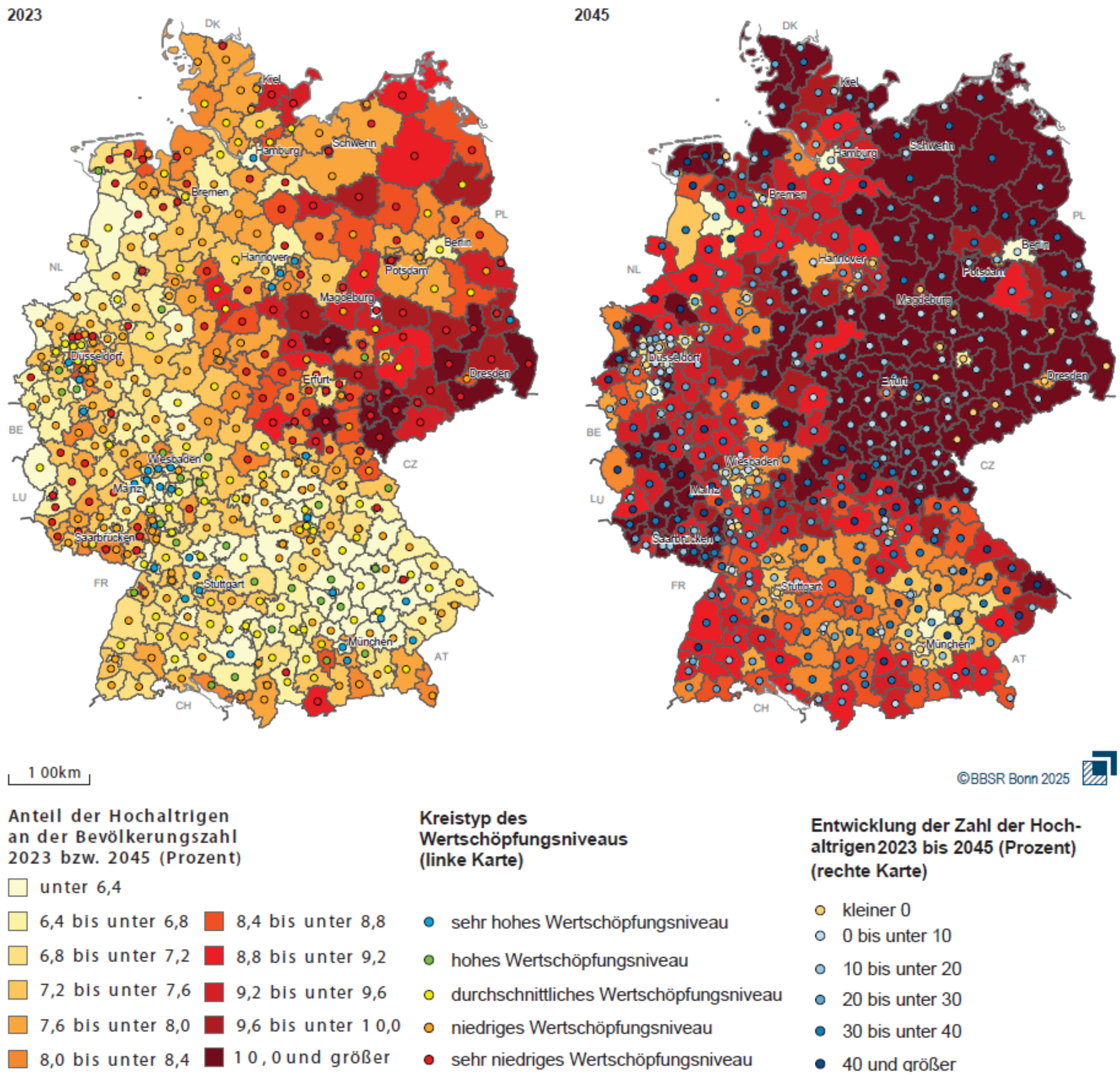

Onlinematerial 2 [33]: Regionstypen des Wertschöpfungsniveaus in Deutschland

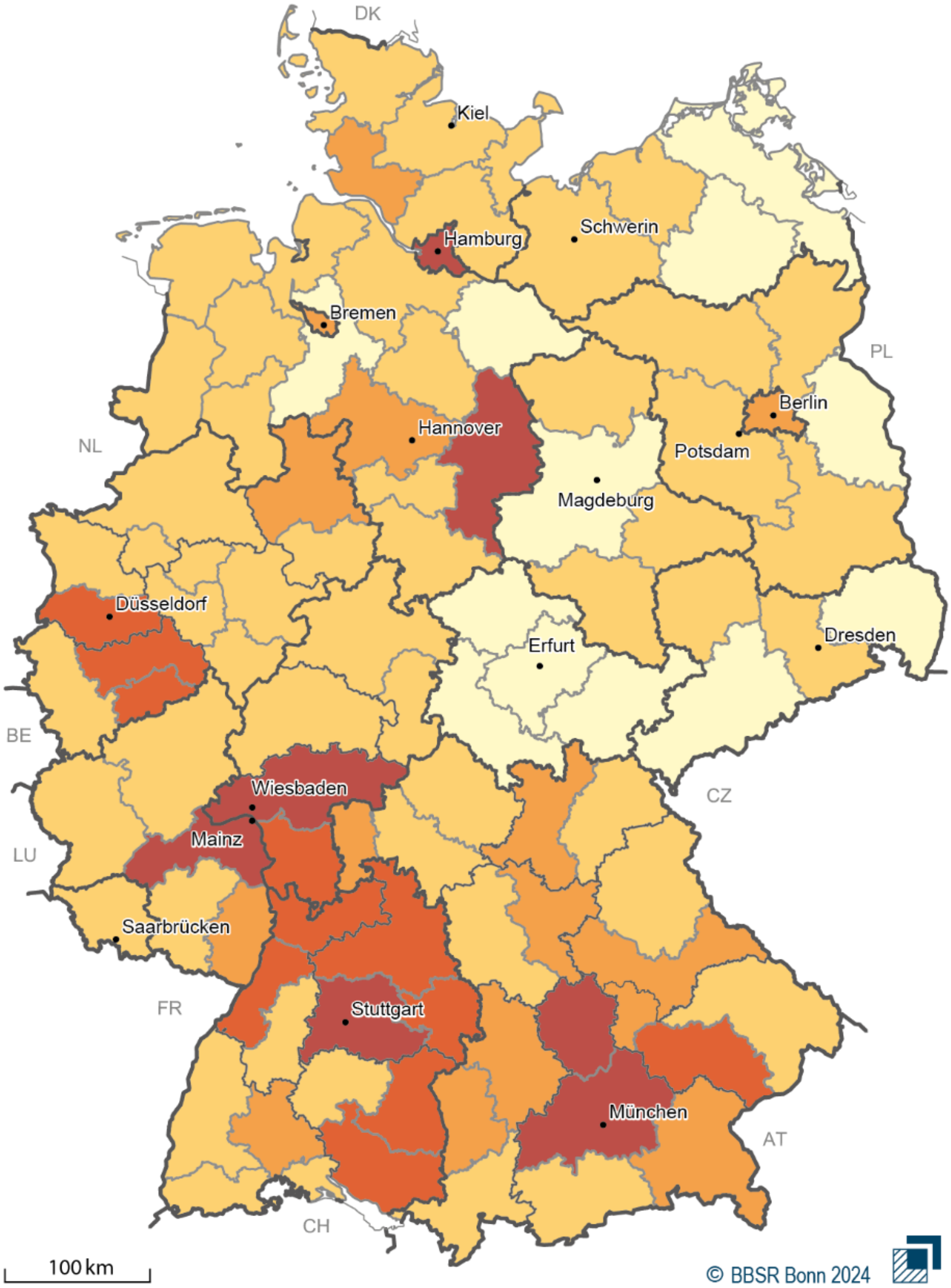

Bruttowertschöpfung je Erwerbstätigem im Durchschnitt der Jahre 2020 und 2021 in 1.000 Euro

- sehr hohes Wertschöpfungsniveau (>= 80,5)
- hohes Wertschöpfungsniveau (73,5 bis unter 80,5)
- durchschnittliches Wertschöpfungsniveau (66,5 bis unter 73,5)
- niedriges Wertschöpfungsniveau (59,5 bis unter 66,5)
- sehr niedriges Wertschöpfungsniveau (< 59,5)

Der Bundesdurchschnitt beträgt rund 70.000 Euro. Das bedeutet wenige Regionen sind für den Großteil der Bruttowertschöpfung verantwortlich. Der Median liegt bei rund 63.600 Euro.

Datenbasis:
Laufende Raumbeobachtung des BBSR
Geometrische Grundlage: VG5000 (Kreise), Raumordnungsregionen
Stand 31.12.2022 © GeoBasis-DE/BKG
Bearbeitung: C. Schlömer
